# Recombinant human TSH stimulated thyroglobulin levels at remnant ablation predict structural incomplete response to treatment in patients with differentiated thyroid cancer

**DOI:** 10.1097/MD.0000000000007512

**Published:** 2017-07-21

**Authors:** Jeonghoon Ha, Min Hee Kim, Kwanhoon Jo, Yejee Lim, Ja Seong Bae, Sohee Lee, Moo Il Kang, Bong Yun Cha, Dong Jun Lim

**Affiliations:** a Division of Endocrinology and Metabolism, Department of Internal Medicine; b Department of Surgery, College of Medicine, The Catholic University of Korea, Korea.

**Keywords:** recombinant human TSH, recurrence, thyroglobulin, thyroid cancer

## Abstract

In patients with differentiated thyroid cancer, stimulated thyroglobulin (sTg) levels after thyroid hormone withdrawal (THW) at remnant ablation (RA) and at 6 to 12 months are known to have good prognostic value. This study aimed to evaluate the prognostic impacts and best cutoff values of sTg levels under recombinant human thyroid stimulating hormone (rhTSH) treatment at RA and at follow-up. A total of 151 patients were enrolled, of whom 77 were followed up with rhTSH-stimulated Tg (rhTSH-sTg) and 74 with THW-stimulated Tg (THW-sTg) at 6 to 12 months after rhTSH-aided RA. Risk stratification, response to treatment (excellent, indeterminate, biochemical incomplete, and structural incomplete response [SIR]), and clinical outcome were accessed by revised American Thyroid Association (ATA) guideline criteria. Seven out of 151 (4.6%) patients were confirmed to have SIR during the median follow-up of 79.0 months; 3 in the rhTSH group and 4 in the THW group. One hundred thirty-two out of 151 (87.4%) patients were confirmed to have excellent response; 68 (51.5%) in the rhTSH group and 64 (48.5%) in the THW group. The cutoff values of sTg for predicting SIR to treatment at rhTSH-aided RA, THW-sTg, and rhTSH-sTg were 4.64 ng/mL (sensitivity 85.7%, specificity 76.4%, negative predictive value [NPV] 99.2%), 2.41 ng/mL (sensitivity 100%, specificity 94.3%, NPV 100%), and 1.02 ng/mL (sensitivity 66.7%, specificity 94.6%, NPV 98.6%), respectively. sTg levels using rhTSH at both RA and follow-up has a high NPV and are as effective as using THW for predicting SIR. The risk classification according to the revised ATA guidelines can be used effectively to supplement rhTSH-aided sTg levels to predict better clinical outcomes.

## 1 Introduction

Differentiated thyroid cancer (DTC) has a good prognosis, with a 10-year survival rate higher than 90%. Many patients with DTC undergo radioiodine remnant ablation (RA) to ablate residual thyroid tissue and to decrease the recurrence rate. Several nonrandomized studies have shown that RA can reduce recurrence rates.^[1,2]^

Serum thyroglobulin (Tg), along with thyroid ultrasonography, is the mainstay for follow-up monitoring of DTC patients after initial treatment.^[3]^ Stimulated serum Tg (sTg) levels measured at RA and 6 to 12 months after initial RA have reliable prognostic value for the prediction of persistent/recurrent disease (PRD).^[4]^ sTg production can be induced by a high thyroid stimulating hormone (TSH) level, which is achieved either by withdrawing thyroid hormone or by the use of recombinant human TSH (rhTSH) thyrotropin. Thyroid hormone withdrawal (THW) causes many acute symptoms of hypothyroidism, including fatigue, weight gain, edema, depressive mood, constipation, and sleep disturbance, whereas rhTSH can be safely applied without concerns about symptoms of hypothyroidism.^[5,6]^

Several studies have demonstrated the predictive value of serum THW-stimulated sTg level measured at RA.^[7–10]^ Kim^[10]^ reviewed 359 patients with papillary thyroid carcinoma who underwent high-dose RA. The authors suggested that a sTg level at RA above 5.22 ng/mL was associated with PRD. Toubeau et al^[7]^ retrospectively reviewed 212 patients with DTC and identified lymph node invasion and sTg at RA as important parameters defining the risk of disease progression.

One recent study reported that the sTg level at rhTSH-aided RA has an independent predictive value for disease-free status at 1 year in the absence of routine prophylactic central neck dissection (CND).^[11]^ Only 1 study has reported that low sTg at rhTSH-aided RA may be a favorable prognostic marker in the presence of prophylactic CND, but this study had only 27 months of follow-up.^[12]^

In the present study, we investigated the predictive value of sTg levels at rhTSH-aided RA for the prediction of structural incomplete response (SIR) according to the revised criteria of American Thyroid Association (ATA) management guideline in DTC patients with prophylactic CND. We also compared the predictive values of sTg at follow-up after applying either THW or rhTSH stimulation.

## 2 Methods

### 2.1 Patients and study design

The study included consecutive patients who presented with DTC without initial distant metastasis between January 2006 and December 2011, and who underwent total thyroidectomy with prophylactic CND followed by rhTSH-aided high-dose RA. sTg at rhTSH-aided RA (A-sTg) and sTg at follow-up, stimulated by either rhTSH (rhTSH-sTg) or THW (THW-sTg) were evaluated for the optimal cutoff value for predicting SIR. Patients were excluded from the analysis for the following reasons: missing data (lack of records for TSH, sTg, or anti-Tg antibody), positive anti-Tg antibody, and insufficient dose of RA.

Risk stratification of the DTC patients was performed by using the revised ATA guidelines.^[4]^ Patients with no local or distant metastasis and no ^131^I uptake outside the thyroid bed on the first post-treatment whole-body scan (WBS) (TxWBS) were defined as low risk. Patients with microscopic invasion of tumor into the perithyroidal soft tissues at initial surgery, cervical lymph node metastasis, ^131^I uptake outside the thyroid bed on TxWBS, or tumor with aggressive histology or vascular invasion were regarded as intermediate risk. Patients with macroscopic tumor invasion or incomplete tumor resection were classified as high-risk.

The institutional review board approved this study (KC15RISI0538).

### 2.2 Laboratory measurements

A-sTg was defined as the serum sTg levels measured under rhTSH stimulation at the time of initial RA, just after total thyroidectomy. A-sTg was measured after 2 consecutive injections of 0.9 mg of rhTSH in the buttock at a 24 hour interval. A-sTg was measured 72 hour after the second rhTSH injection. sTg at follow-up was obtained 6 to 12 months after RA. rhTSH-sTg at follow-up was measured by using the same protocol as for A-sTg measurement during RA. THW-sTg was measured at least 2 weeks after cessation of T3, which had replaced thyroxine 2 weeks before that. Serum Tg levels were measured with an immunoradiometric assay (IRMA) kit with a functional sensitivity of 0.7 ng/mL (CIS Bio International, Codolet, France). Anti-Tg antibody was measured with a competitive radioimmunoassay kit (ZenTech, Angleur, Belgium). Anti-Tg antibody was considered positive when the value was above 70 IU/mL. TSH levels were detected with an IRMA kit (Beckman Coulter, Brea, CA) with a detection limit of 0.0025 mIU/L.

### 2.3 Determination of disease status

According to revised ATA guidelines, the criteria for classifying response to treatment were as follows^[4]^:

(1) Excellent response: no evidence by imaging studies and either suppressed Tg < 0.2 ng/mL or stimulated Tg < 1 ng/mL.
(2) Biochemical incomplete response: no evidence of recurrence on imaging studies and suppressed Tg ≥ 1 ng/mL in the absence of anti-Tg Ab or stimulated Tg ≥ 10 ng/mL in the absence of anti-Tg Ab or rising anti-Tg Ab levels.
(3) SIR: structural or functional evidence of disease with any Tg level with or without anti-Tg Ab.
(4) Indeterminate response: nonspecific findings on imaging studies, nonstimulated Tg detectable (but <1 ng/mL), stimulated Tg detectable (but <10 ng/mL) or anti-Tg Ab stable, or declining in the absence of structural or functional disease.

Identifiable lesions were found by imaging methods, including diagnostic WBS (DxWBS), computed tomography (CT), or positron emission tomography (PET)/CT.^[10]^ In this study, SIR is used in the same sense as PRD formerly used in the previous guidelines.

### 2.4 Statistical analysis

In the analysis of categorical variables, a χ^2^ test or Fisher exact test was applied. The Mann–Whitney *U* test or independent *t* test was used where appropriate to compare continuous variables. Values with a normal distribution were expressed as mean ± standard deviation, and values with a non-normal distribution were expressed as median and range. Tg cutoff values were determined by receiver operating characteristic (ROC) analysis to determine the Tg levels that maximized the sum of sensitivity and specificity. Diagnostic performance parameters of rhTSH-sTg, including sensitivity, specificity, positive predictive value, and negative predictive value (NPV), were evaluated on the basis of the cutoff values obtained by ROC analysis. SPSS for Windows, version 12.0 (SPSS Inc., Chicago, IL) was used for all the statistical analyses.

## 3 Results

### 3.1 Baseline clinical characteristics of study population

Of 183 patients who received high dose RA under the use of rhTSH, we excluded those who first, had missing data (lack of Day 2 data (n = 2), lack of Day 5 data (n = 6), lack of both Day 2 and Day 5 data (n = 5); second, had high anti-Tg antibody levels (n = 4); third, lost to follow-up (n = 13); and fourth, underwent ablation with >150 mCi or <100 mCi radioiodine (n = 2). After excluding 32 patients, the study included 151 patients who underwent rhTSH-aided RA, 115 of whom were female (76.2%) and whose mean age at diagnosis was 48.9 ± 12.4 years. The median follow-up duration was 79.0 months (range 55.4–91.4 months). Most patients were diagnosed with papillary carcinoma and 1 patient with follicular carcinoma. Among the papillary carcinomas, the majority were of the classic type and the follicular variant was found to be about 12% of the total. Tall cell variant and Warthin-like variant were also identified in each case. One hundred thirty-two (87.4%) patients were confirmed to have excellent response. SIR was identified in 7 patients, 5 of which were high risk by ATA risk stratification but none were low risk. The detailed baseline characteristics of the study population are summarized in Table 1.

**Table 1:**
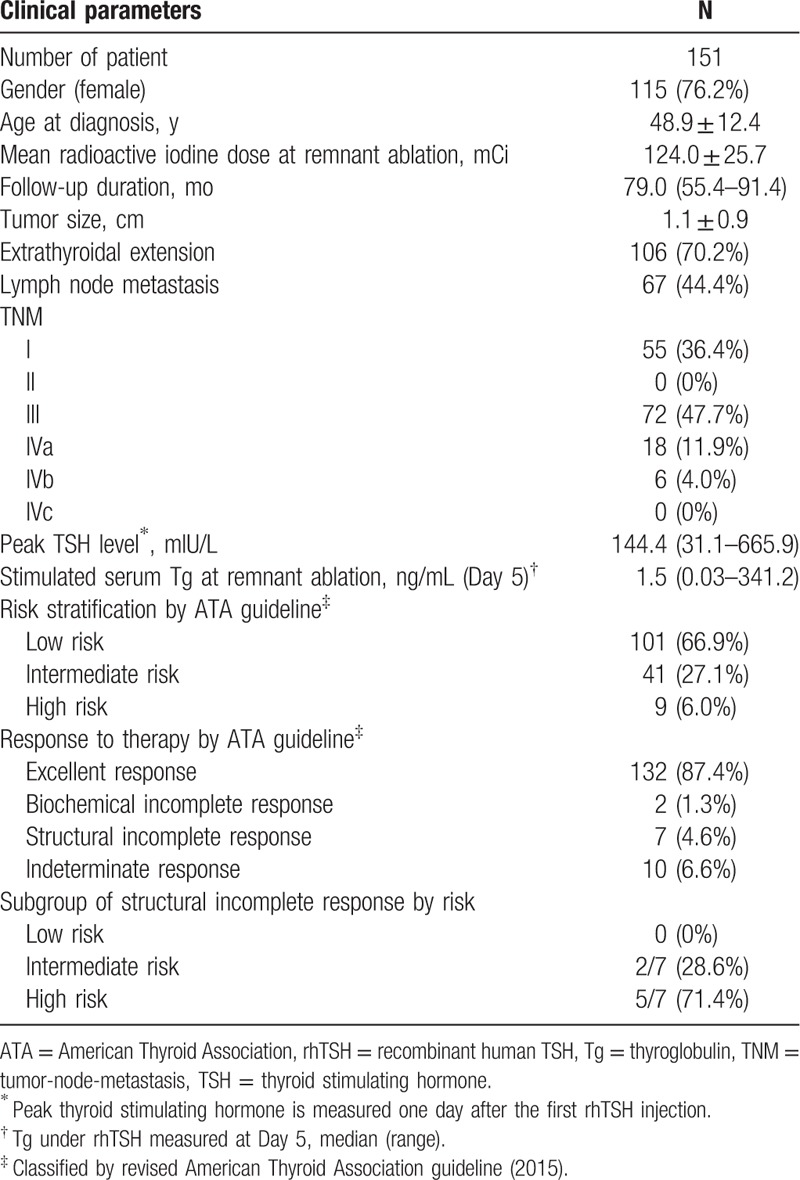
Baseline clinical characteristics of study population.

### 3.2 Clinical characteristics of study subjects according to TSH stimulation method at 6 to 12 month follow-up

**Figure 1:**
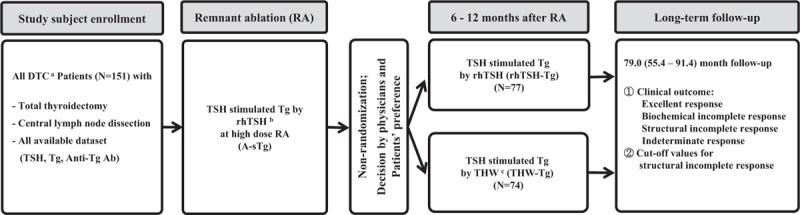
Time flow for management of study subjects. ^a^DTC, Differentiated thyroid cancer; ^b^rhTSH, recombinant human TSH; ^c^THW, thyroid hormone withdrawal.

Of 151 patients enrolled, 77 (51.0%) underwent rhTSH-sTg measurement and 74 (49.0%) underwent THW-aided Tg measurement at 6 to 12 month follow-up (Fig. 1). The clinical data and characteristics of both groups are summarized in Table 2; sex, age at diagnosis, the mean radioiodine dose at RA, follow-up duration, tumor size, tumor-node-metastasis stage, and distribution of risk stratification did not differ significantly between the 2 groups. Three patients undergoing rhTSH-aided follow-up and 4 patients undergoing THW-aided follow-up were confirmed to have SIR. Response to treatment assessed according to revised ATA classification did not differ between both groups (*P* = 0.977).

**Table 2:**
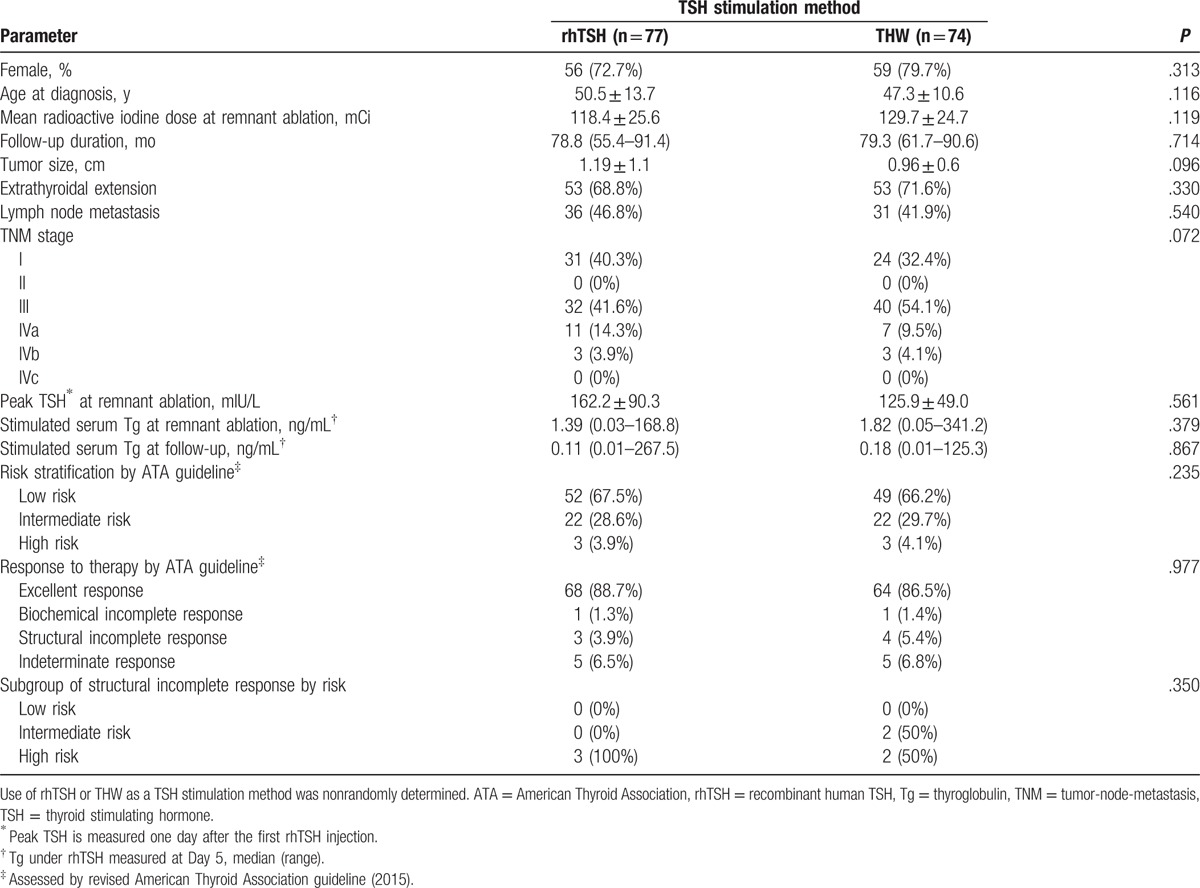
Clinical characteristics of study subjects according to different TSH stimulation methods at 6 to 12 month follow-up.

### 3.3 Predictive value of sTg levels at the time of rhTSH-aided RA and at first follow-up with rhTSH or THW

Of the patients who underwent rhTSH-aided RA, 7 (4.6%) patients were confirmed to have SIR. The ROC analysis showed that the optimal cutoff value for the A-sTg that predicted SIR was 4.64 ng/mL (area under curve = 0.898). This cutoff value produced a sensitivity of 85.7%, specificity of 76.4%, and NPV of 99.2% (Table 3). Of the 74 patients who underwent THW-aided follow-up at 6 to 12 months, 4 (5.4%) were confirmed to have SIR. ROC curve analysis showed 2.41 ng/mL (area under curve = 0.998) as the best cutoff value with a sensitivity of 100%, specificity of 94.3%, and NPV of 100% (Table 3). Similar analyses for the patients with rhTSH-aided follow-up at 6 to 12 months showed 1.02 ng/mL (area under curve = 0.941) with a sensitivity of 66.7%, specificity of 94.6%, and NPV of 98.6% (Table 3).

**Table 3:**
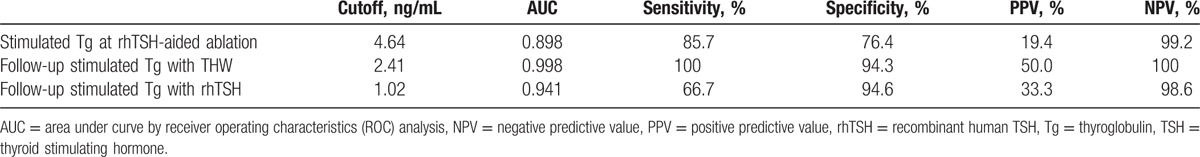
Comparison of TSH stimulated thyroglobulin cutoff values and diagnostic performances to predict structural incomplete response at the time of remnant ablation and at first follow-up with rhTSH or thyroid hormone withdrawal.

### 3.4 Breakdown of SIR cases and their association with ATA risk stratification

The detailed characteristics of the 7 patients confirmed as having SIR are described in Table 4. Patients 1 to 3 underwent rhTSH-aided follow-up and patients 4 to 7 underwent THW-aided follow-up. Patients 3, 4, and 5 were confirmed to have pulmonary metastasis and patients 1, 2, 6, and 7 to have cervical lymph node metastasis. Five out of 7 patients were in the high risk group and the other 2 were in the intermediate group according to the ATA risk stratification, but none in the low risk group. SIR patients showed Tg levels higher than cutoff values, both at ablation and at 6 to 12 month follow-up (Table 5), except 1 (patient 1). In patient 1, the A-sTg at Day 5 was 1.8 ng/mL. The patient was classified as high-risk group due to the presence of macroscopic extrathyroidal extension and extensive vascular invasion. The patient received RA with 150 mCi radioiodine, and TxWBS revealed that focal radioiodine uptake was present in the anterior neck. After 6 months, the rhTSH-sTg was 0.3 ng/mL, which is below the functional sensitivity of our institution and DxWBS showed no remnant thyroid tissue or functioning metastasis. Three months later, an 8-mm diameter lymph node enlargement was detected in the right thyroid bed (Level VI). Biopsy confirmed recurrent papillary thyroid carcinoma and radiofrequency ablation was performed for the recurrent lesion. Patient 3 (high risk) had strikingly high levels of Tg at RA, A-sTg (168.8 ng/mL), and rhTSH-sTg (267.5 ng/mL). A large tumor burden was detected in the cervical lymph node area (levels III, IV, V, VI) during preoperative evaluations including neck CT and PET/CT. Radioiodine uptake in the lung was not detected at RA. The patient was finally confirmed to have lung metastasis 19 months after the surgery.

**Table 4:**
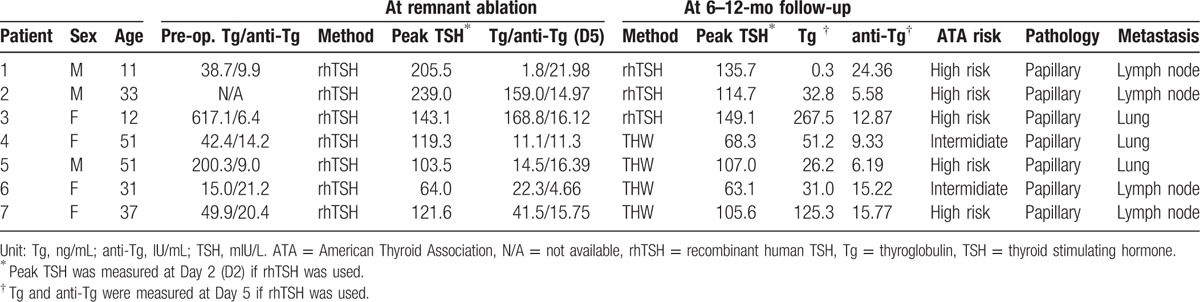
Clinical characteristics of 7 patients with structural incomplete response.

**Table 5:**
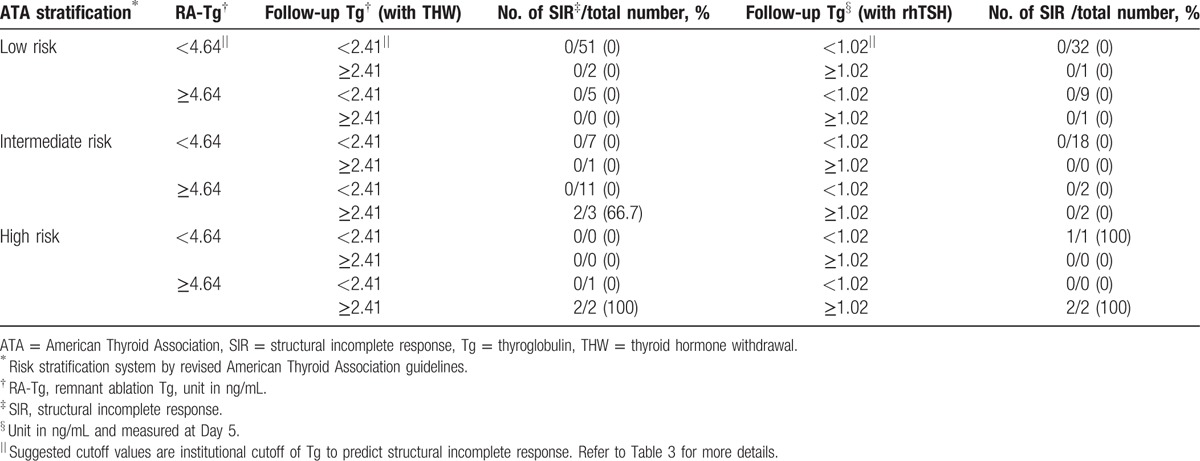
Breakdown of structural incomplete response cases in study population by ATA risk stratification, ablation Tg, and follow-up Tg.

## 4 Discussion

Since rhTSH was introduced to thyroid medicine, its use has been extended from a diagnostic tool replacing THW-aided sTg to a therapeutic option for supporting a safe and effective RA.^[13–15]^ From an economic point of view, it is known to be a cost-effective method of TSH stimulation. Sohn et al^[16]^ concluded that the use of rhTSH before RA was a cost-effective alternative to endogenous hypothyroid stimulation.

In recent years, several reports have investigated the predictive value of A-sTg at the time of initial THW-aided RA but few publications are available for the predictive value of A-sTg at rhTSH-aided RA. In the present study, it was possible to examine the diagnostic performance of A-sTg in DTC patients undergoing prophylactic CND.

In our previous study, we determined the cutoff value for sTg under THW-aided ablation for prediction of PRD, which was renamed to SIR, the most similar description in the revised guideline and concluded that the cutoff value determined by ROC curve analysis was 5.22 ng/mL.^[10]^ Ciappuccini^[17]^ suggested an optimal A-sTg cutoff level of 2.8 ng/mL for predicting PRD, although a major limitation of that study was that A-sTg was measured 2 days after the first rhTSH injection (Day 3), necessarily resulting in lower Tg levels. Recent ROC reanalysis of previous serial Tg data to determine the appropriate timing of sTg measurement identified Day 5 as the time at which most patients had peak Tg levels,^[18]^ and the US Food and Drug Administration specified that sTg should be measured 3 days after the second injection of rhTSH (Day 5).^[5]^ Our study followed this approved protocol for sTg measurement. Melo et al assessed disease status at 1 year and found that a higher sTg level at the time of rhTSH-aided ablation was associated with PRD. They suggested 7.2 ng/mL as the cutoff value for rhTSH-aided sTg. But sTg was even measured 3 days after the RA, without routine central lymph node dissection, resulting in a higher sTg cutoff level of 7.2 ng/mL.^[19]^

Recently, Moon et al^[12]^ proposed that the optimal cutoff value of sTg to predict PRD at rhTSH-aided RA was 1.79 ng/mL when prophylactic CND (either ipsilateral or bilateral depending on patients’ disease status) was applied. In their study, however, the authors were aware that every study subject with PRD was not pathologically or cytologically proven. Without structural evidence from histopathologic evaluation, the incidence of PRD may appear to be increased (11.9%), eventually affecting cutoff values. It is expected that prophylactic CND may remove any remnant tissue, and hence reduce sTg levels at the time of ablation and recurrence rate during follow-up.

In our study, we followed up for about 6 years patients with RA after rhTSH, and we were able to confirm the treatment response according to the revised ATA guidelines. At the end of follow-up, 132 patients (87.4%) had excellent response, and 7 (4.6%) had SIR. Patients with biochemical incomplete response and indeterminate response were 2 (1.3%) and 10 (6.6%), respectively. At follow-up, there was no significant difference in treatment response whether under rhTSH-aided or THW-aided. In recent study by Pitoia et al^[20]^, 62.7% of patients under rhTSH-aided RA showed no evidence of disease and 15.7% of patients showed SIR. Although the characteristics of study population was different and the number of high-risk patients was relatively smaller in our study, it is likely that prophylactic CND yields better response profiles, probably at the cost of increased incidence of complications like hypoparathyroidism in our study.

It is useful to show that most patients are less likely to have SIR to treatment through the confirmation of high NPV if there is little chance of recurrence as in our study. In the present study, the A-sTg cutoff of 4.64 ng/mL at RA has a high NPV (99.2%), indicating that SIR is observed in less than 1% of patients whose A-sTg level is lower than cutoff. The cutoffs of rhTSH-sTg and THW-sTg also have high NPV (98.6% and 100%, respectively), which means that both stimulation methods were excellent and this concept was already reflected in the revised ATA guidelines.^[4]^

Combining the proposed cutoff value and the risk stratification by revised ATA guidelines, it can be used as an effective tool for predicting SIR. As in Table 5, A-sTg was found to be an important prognostic tool for predicting SIR. Once the A-sTg level was less than the cutoff value, SIR was not observed in low and intermediate-risk groups. Furthermore, in patients classified as low-risk group, SIR was not observed regardless of the values of A-sTg, rhTSH-sTg, or THW-sTg. However, in the high-risk group, sTg at follow-up, either rhTSH-sTg or THW-sTg, can be significant once A-Tg is greater than cutoff. For example, if A-Tg and rhTSH-sTg are higher than the cutoff in the high risk group, it is judged explicitly as SIR, and more thorough evaluation, including diagnostic WBS or chest CT scan in addition to neck ultrasonography should be done. As in patient 1 of our study, if the patient is in high-risk group, cautious systemic and pathologic review of the possibility of SIR is warranted even if sTg levels were below the cutoff value at rhTSH-based ablation.

Our study has several limitations. First, it did not include an appropriately matched control group of patients who underwent THW-aided RA. These patients might show higher sTg values than our study subjects but no direct comparison could be made. Second, because our study population had a very low rate of recurrence compared with those reported in other studies, it might have been difficult to adequately identify the cutoff value for predicting SIR to treatment. Strict management protocols, including the use of prophylactic CND and high dose RA, could have influenced this low rate of SIR to treatment (4.6% over the follow-up period of 79.0 months). Another limitation is related to patient baseline characteristics. Of the 7 patients with metastasis, 2 were less than 15 years old. We hypothesize that the pediatric patients had limited access to a thyroid sonogram, which resulted in the late diagnosis of thyroid cancer with an increased tumor burden. We need to take special note of pediatric patients in further investigations about the characteristics of metastatic thyroid cancer in pediatric patients.

Despite its limitations, our study has important clinical implications. This study classified treatment response according to revised ATA guidelines and investigated the effectiveness of rhTSH-sTg at both RA and follow-up in the setting of prophylactic CND over more than 6 years of follow-up. We found that Tg cutoff level of 4.64 ng/mL under rhTSH aided RA had a NPV of 99.2%, indicating less than 1 percent of SIR with Tg lower than 4.64 ng/mL. At follow-up, sTg levels, either by rhTSH or by THW, also have a high NPV for predicting SIR in DTC patients with rhTSH-based RA. We also found that both ATA risk stratification and the proposed Tg cutoff were important in evaluating patients. If the ATA risk is high, SIR occurred even though the rhTSH-Tg value was low. On the other hand, at intermediate risk, SIR was found to occur when Tg was higher than cutoff. SIR was not observed in patients with low risk. Taken together, ATA risk stratification and sTg value play a complementary role, helping clinicians to predict SIR efficiently, and helping to apply different follow-up protocol to each patient. For example, if ATA risk is low or intermediate but A-Tg is below cutoff, it may even be possible to omit follow-up stimulation Tg measurement at 6 to 12 months after surgery. However, as in our case, we still recommend careful monitoring of patients for SIR if a patient is considered high risk by ATA guidelines but showed low sTg levels.
